# Machine learning and near-infrared fusion-driven quantitative characterization and detection of protein content in maize kernels

**DOI:** 10.3389/fnut.2025.1719661

**Published:** 2025-12-17

**Authors:** Yang Yu, Yongkun Qiao, Chenlong Fan, Man Dong, Ke Cao

**Affiliations:** 1 College of Agricultural Engineering, Jiangsu University, Zhenjiang, China; 2 Key Laboratory for Theory and Technology of Intelligent Agricultural Machinery and Equipment, Jiangsu University, Zhenjiang, China; 3 College of Mechanical and Electronic Engineering, Nanjing Forestry University, Nanjing, China

**Keywords:** near-infrared spectroscopy, maize powder, protein content, machine learning, detection

## Abstract

This study aims to develop a rapid and non-destructive method for determining protein content in maize using near-infrared spectroscopy (NIRS). To mitigate the effects of surface irregularities and uneven protein distribution in whole kernels on spectral measurements, maize powder was used as the test material to enhance the uniformity and stability of spectral signals. A total of 90 maize powder samples were collected from major production regions across China, and a custom NIRS acquisition system was constructed. To optimize the spectral data, eight preprocessing methods—including Multiplicative Scatter Correction (MSC), Standard Normal Variate (SNV), First Derivative (1D), Savitzky–Golay smoothing (S–G), and their combinations—were systematically evaluated. Subsequently, traditional machine learning models (Partial Least Squares Regression, PLSR; Support Vector Machine, SVM) and deep learning models (ResNet-18, Transformer) were developed to predict protein content, and their performances were compared. Results indicated that the combined preprocessing strategy of First Derivative and Multiplicative Scatter Correction (1D + MSC) was the most effective. Among the models, PLSR demonstrated the best predictive performance, and traditional chemometric methods showed greater practical utility compared to deep learning models. To further enhance model efficiency, four feature wavelength selection methods—Partial Least Squares Regression Coefficients (PLSRC), Competitive Adaptive Reweighted Sampling (CARS), Successive Projections Algorithm (SPA), and Uninformative Variable Elimination (UVE)—were applied. It was found that the PLSR model combined with the Successive Projections Algorithm (SPA) yielded the optimal performance, achieving a validation set correlation coefficient (*R*_p_) of 0.927, a root mean square error of prediction (RMSE_P_) of 0.301, and a residual predictive deviation (RPD) of 2.502, along with the fastest computational speed. This study provides a reliable technical solution and theoretical foundation for the rapid and non-destructive detection of protein content in maize, while also validating the advantage of using powdered samples in improving the accuracy of NIRS detection.

## 1 Introduction

Global demand for grain continues to rise. Maize, as a crucial crop for food, feed, and industrial raw materials, has its yield and quality directly impacting both food security and the agricultural economy (1). The nutritional value and processing suitability of maize largely depend on its key component contents, such as protein, starch, and fat (2). Among these, protein content is a vital indicator for assessing maize quality. It influences not only its nutritional value as food but also its effectiveness in animal feed and deep-processing applications (3). The quality of cash crops such as corn may deteriorate during the post-production process (harvest, storage, transportation) (4). Therefore, establishing efficient and accurate methods for detecting maize protein content is significant for optimizing maize breeding, processing, and market distribution. Traditional protein quantification methods, such as the Kjeldahl method, spectrophotometry, and the Dumas method, are reliable (5–8). However, they are inefficient and involve complex procedures, making it difficult to meet the rapid detection needs of modern agriculture and food processing. Hence, researching high-efficiency, low-cost rapid detection technologies for maize protein is highly valuable for enhancing quality control across the maize industry chain.

In recent years, intelligent detection technologies like near-infrared spectroscopy (NIRS) and machine learning have advanced rapidly. Owing to their speed and environmental friendliness, they show broad application prospects in agricultural product quality analysis (9). Near-Infrared Reflectance spectroscopy (NIRS) is a fast, non-destructive, reliable, and eco-friendly detection technique. It has been successfully used to determine protein content in various feed materials (10–12). Lin et al. developed a sensor based on NIRS characteristic wavelengths for rapid moisture detection in paddy rice, achieving precise online measurement with a coefficient of determination (*R*^2^) of 0.936 and a standard error of estimation (SEE) of 23.44 (13). Tian et al. established a model for detecting crude protein content in brown rice using NIRS (14). Their model achieved a coefficient of determination (*R*^2^) of 0.9185, a cross-validation *R*^2^ (*R*^2^_cv_) of 0.8876. Xu et al. (15) analyzed the feasibility of using NIRS combined with chemometrics to detect protein in maize. The protein regression model they developed met the requirements for maize component detection. NIRS technology works by detecting the absorption or reflection of near-infrared light by a sample to obtain its characteristic spectral information. This spectral data is then processed to determine chemical information such as protein and moisture content. NIRS is now widely used in grain quality detection and demonstrates excellent analytical performance.

While NIRS performs well in detecting maize protein content, it still has limitations. Current research mainly focuses on protein detection in whole maize kernels. The surface roughness and morphological variations of whole kernels can cause light scattering effects. Additionally, uneven protein distribution within a single kernel can affect measurement repeatability (16, 17). To address these issues, using maize powder can reduce the impact of particle differences, improve spectral uniformity, increase light penetration depth, and enhance the spectral response. The complexity of NIRS also poses a challenge for data interpretation. NIRS are highly complex, consisting of many overlapping peaks (known as multicollinearity) due to overtones, combination bands, and vibrations, resulting in broad spectral bands (18). Redundant interference and unnecessary collinearity can weaken model performance (19). Therefore, extracting a limited but sufficient number of characteristic wavelengths for specific chemical components can improve computational efficiency, enhance model performance, increase practical value, and facilitate further exploration of underlying information. Thus, researching NIRS quantitative detection technology for protein content in maize powder is highly significant for advancing quality detection in maize and other grains. However, current research exhibits notable deficiencies in the following aspects. Firstly, systematic spectral analysis focusing on maize powder—a sample form that effectively enhances spectral consistency—remains insufficient, lacking optimization and comparison of different preprocessing methods and modeling strategies. Secondly, and more importantly, building upon this superior data foundation, there is an even greater lack of in-depth investigation into the potential and applicability of deep learning models in this specific context, as well as a comprehensive performance comparison between these advanced algorithms and traditional chemometric methods.

Consequently, this study proposes a method for detecting protein content in maize powder based on NIRS. We collected and analyzed the NIRS information of maize powder. Eight different preprocessing methods were applied and their effects on the spectral data were compared. Four models were built based on full wavelengths: Partial Least Squares Regression (PLSR), Support Vector Machine (SVM), Residual Network (ResNet-18), and Transformer. This allowed a comparison of prediction performance between traditional machine learning and deep learning methods. Furthermore, four feature variable selection methods were employed: Partial Least Squares Regression Coefficients (PLSRC), Competitive Adaptive Reweighted Sampling (CARS), Successive Projections Algorithm (SPA), and Uninformative Variable Elimination (UVE). By optimizing the protein content prediction model, this study aims to provide a reliable technical solution for the rapid, non-destructive detection of protein content. It also seeks to offer a theoretical basis for quality testing and control in maize.

## 2 Materials and methods

### 2.1 Maize powder sample collection

**Figure 1:**
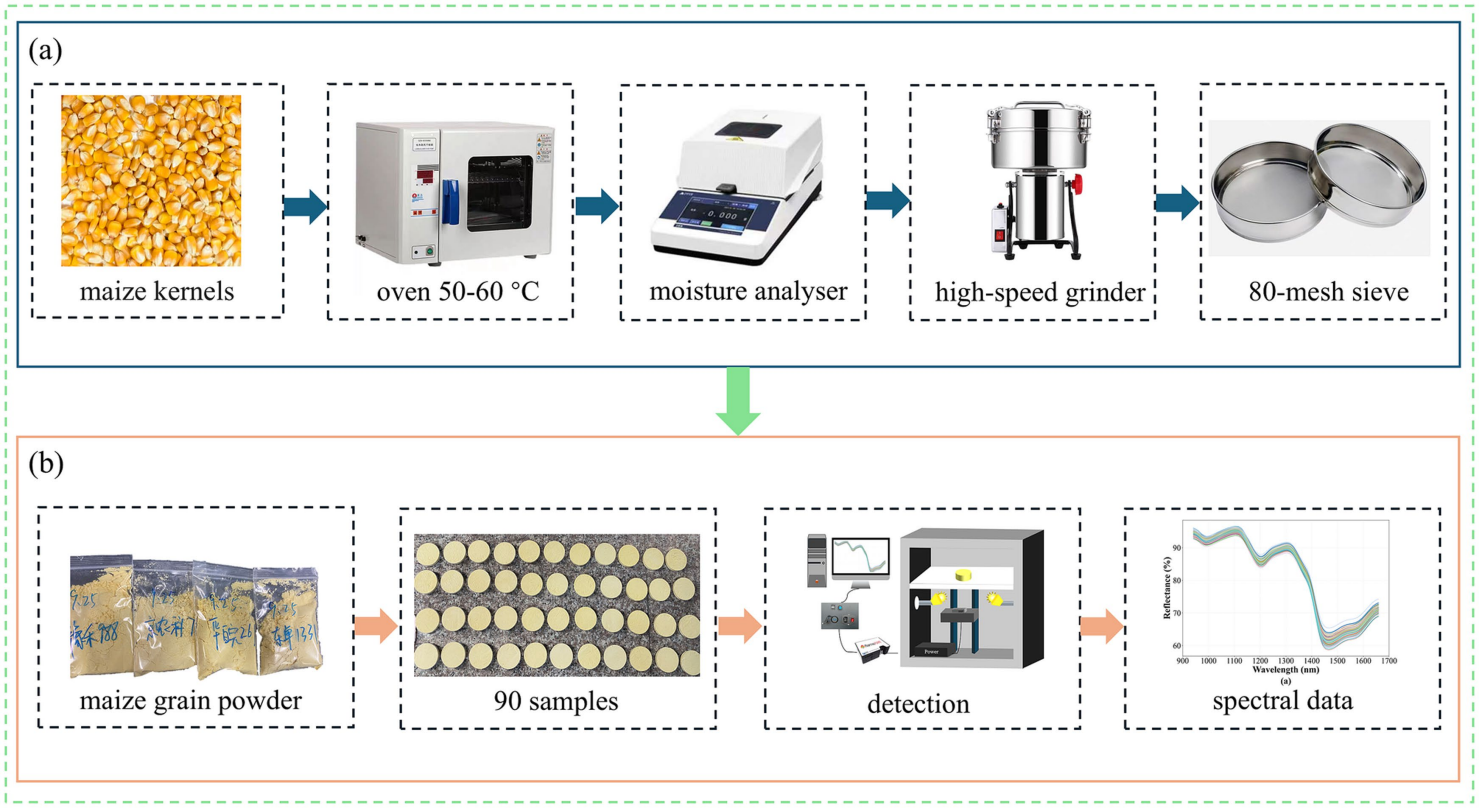
Sample Collection. **(a)** Maize powder preparation process. **(b)** Near-infrared spectral data acquisition process for corn powder.

To ensure the dataset’s broad representativeness and generalisability, and to minimize the gap between research conditions and practical application, maize grain powder from different regions and varieties were collected. These represented five major production areas in China: North China, Northeast China, Southwest China, Northwest China, and the Huang-Huai-Hai region. Before grinding, the kernels underwent washing and drying. First, fresh kernels were selected and cleaned to remove impurities. After washing, the kernels were dried in an oven (GZX-9140MBE, Shanghai, China) set at 50–60 °C. The moisture content of the dried kernels was measured using a moisture analyzer (XIUILAB MB27, Shanghai, China) and confirmed to be 13.05%. This meets the Chinese national standard requirement for dried maize kernels, which specifies a moisture content between 13 and 14%. This drying process effectively reduces interference from hygroscopicity during the subsequent NIRS-based protein detection. Subsequently, the dried kernels were ground into powder using a high-speed grinder (AZL 4500A, Jinhua, China). The resulting powder was passed through an 80-mesh sieve to obtain a uniform, fine powder suitable for subsequent physico-chemical analysis. A total of 90 samples were prepared for this study. For each sample, three NIRS were collected from three random measurement points. The average of these three spectra was used as the final spectral data for that sample. The sample preparation process for maize powder and the near-infrared spectral data acquisition process are shown in Figure 1.

### 2.2 Design of the near-infrared detection system

**Figure 2:**
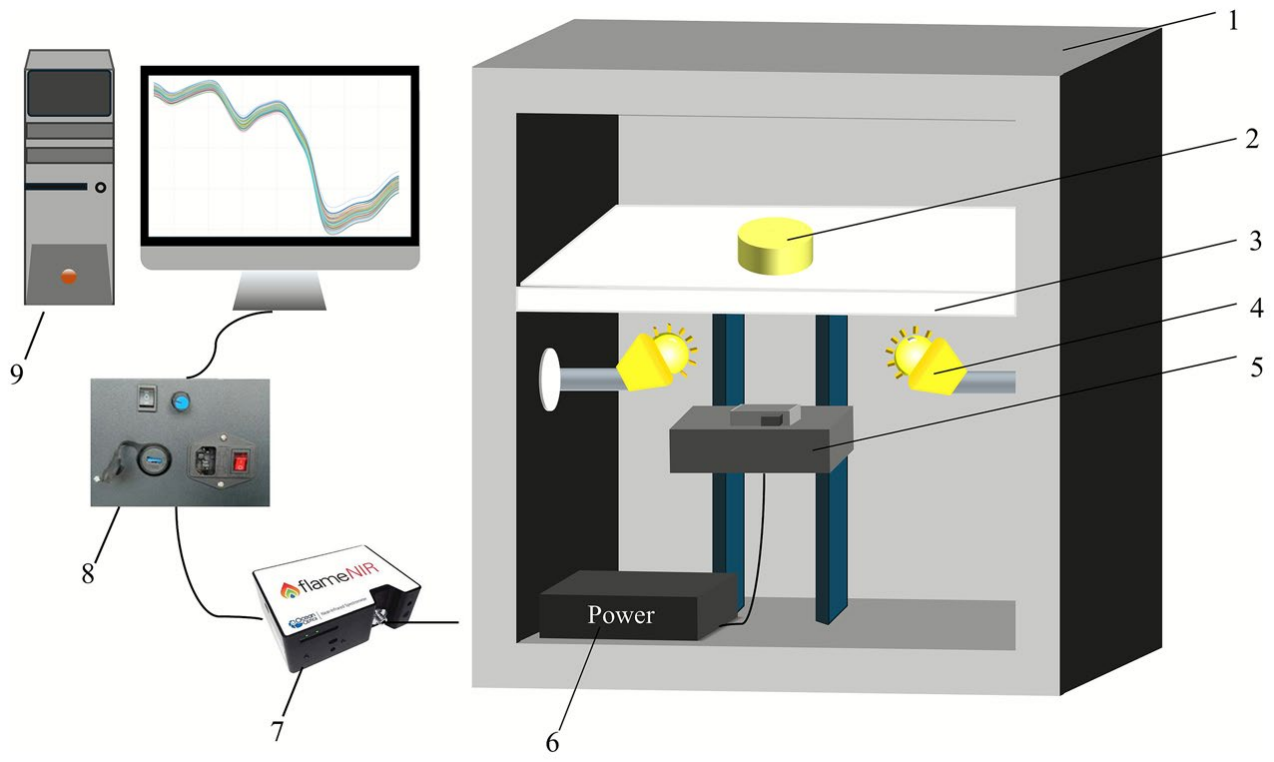
Near-infrared spectral acquisition system. 1. Dark box; 2. Samples; 3. Sample stage; 4. Light source; 5. Optical fiber and lifting platform; 6. Power supply; 7. Spectrometer; 8. Controller; 9. Computer.

The design of a suitable near-infrared detection system is crucial for acquiring spectral data from maize powder. The detection system designed in this study primarily consists of an optical fiber, a light source, a sample stage, a spectrometer, a controller, and a computer, as shown in Figure 2. We selected the FLAME-NIR-INTSMA25 spectrometer (Ocean Optics, Dunedin, FL, United States), which operates within a wavelength range of 940 to 1,660 nm. By avoiding the visible light spectrum (typically 380–780 nm), this system effectively minimizes the potential influence of the maize powder’s color on the overall prediction accuracy of protein content.

### 2.3 Spectral preprocessing

During the collection of sample spectral information, the detection system captures not only the spectral data of the maize powder but also various unwanted signals. These can include electrical noise, stray light, sample background, and other irrelevant external interference. Applying appropriate preprocessing to the spectral data helps to reduce this noise and improve the signal-to-noise ratio. This step is crucial for the subsequent analysis of the spectra and the development of robust models. Common spectral preprocessing algorithms include derivative methods, smoothing, Multiplicative Scatter Correction (MSC), and Standard Normal Variate (SNV) transformation (20–23). Based on the characteristics and information content of the near-infrared spectra from maize powder, this study selected eight preprocessing methods. These are: Multiplicative Scatter Correction (MSC), Standard Normal Variate (SNV), First Derivative (1D), Savitzky–Golay (S–G) smoothing, 1D + SNV, 1D + MSC, SG + SNV, and SG + MSC.

#### 2.3.1 Multiplicative scatter correction (MSC)

Multiplicative scatter correction (MSC) effectively eliminates spectral differences caused by varying scattering levels, thereby improving the signal-to-noise ratio of the data. MSC works by aligning each sample spectrum to a reference spectrum (usually the mean spectrum of all samples) through a linear transformation. This process removes both additive offsets and multiplicative scaling effects (changes in slope) caused by scattering in powdered or granular samples. Consequently, it reduces physical scattering variations while preserving the shapes of the chemical absorption bands. After MSC processing, the physical scattering differences between all sample spectra are significantly suppressed, primarily retaining absorption features related to chemical composition. This greatly enhances the robustness and accuracy of subsequent qualitative or quantitative analysis models. First, the average value $\bar{R}$ of n spectral data points is calculated. The calculation formula is as follows (Equation 1):


$$\bar{R}=\frac{1}{n}\;\sum_{i=1}^{n}R_{i}$$ (1)

Then, a univariate linear regression is performed between R and $\bar{R}$ to calculate the bias (offset) $\mathrm{Bia}s_{i}$ and the gain (slope) $\mathrm{Gai}n_{i}$ for each individual spectrum (Equation 2).


$$R_{i}=\mathrm{Gai}n_{i}\;\bar{R}+\mathrm{Bia}s_{i}$$ (2)

After the correction process, the final obtained spectral data is $\;R_{i,\mathrm{MSC}}$ (Equation 3).


$$\begin{array}{c} R_{i,\mathrm{MSC}}=\frac{R_{i}\;-\mathrm{Bia}s_{i}\;}{\mathrm{Gai}n_{i}} \end{array}$$ (3)

#### 2.3.2 Standard normal variate (SNV)

Standard Normal Variate (SNV) is an important method widely used in the preprocessing of spectral data. Its core principle involves standardizing each individual spectral curve through zero-meaning and unit variance scaling. This process effectively eliminates systematic errors introduced by differences in the physical characteristics of samples. These include additive baseline offsets and multiplicative scattering effects caused by uneven particle size distribution, inconsistent sample packing density, and variations in optical path length. By mitigating variations arising from these non-chemical factors, SNV enhances the spectral features related to the material’s chemical composition itself. Consequently, it improves the signal-to-noise ratio of the spectral data in quantitative analysis. This method lays an important foundation for establishing accurate qualitative or quantitative analytical models. The calculation formula is as follows (Equation 4):


$$\begin{array}{c} Z=\frac{X-\mu }{\sigma } \end{array}$$ (4)

Where Z represents the standard normal variable (following a normal distribution with a mean of 0 and a standard deviation of 1); X represents the original normal variable; μ represents the mean of the original normal distribution; and σ represents the standard deviation of the original normal distribution.

#### 2.3.3 First derivative (1D)

First derivative preprocessing serves to accentuate the peaks and troughs within a spectrum. This makes the spectral features more distinct and aids in identifying and analyzing subtle variations in the spectral data. Furthermore, this method effectively eliminates baseline shifts and background interference present in the spectrum. It results in a more stable baseline, thereby enhancing the accuracy of the subsequent analysis.

#### 2.3.4 Savitzky–Golay convolution smoothing (S–G)

Savitzky–Golay convolution smoothing (S–G) performs a least-squares fit using a low-order polynomial within a moving window on the original spectrum. It replaces the central point’s value with the value (or derivative) from the fitted polynomial. This method reduces noise while striving to preserve the original peak shapes and positions, thereby improving the signal-to-noise ratio of the spectrum and the robustness of subsequent models. Based on the spectrometer’s pixel characteristics and empirical practice, this study selected a Savitzky–Golay smoothing window size of 7 (i.e., fitting within a window of 7 data points) and a polynomial order of 2.

This study thoroughly investigated the characteristics of various spectral preprocessing methods and systematically analyzed their effects when applied individually. To further enhance preprocessing efficiency and accuracy, we innovatively combined some methods to form a series of composite preprocessing strategies. In combined preprocessing methods, first-order derivative (1D) or Savitzky–Golay (SG) smoothing is applied first, followed by Standard Normal Variate (SNV) and Multiplicative Scatter Correction (MSC) processing. This can effectively ensure the validity of scatter correction by SNV and MSC. Meanwhile, derivative processing amplifies high-frequency noise. If SNV or MSC is performed first, followed by derivative processing, this amplified noise will be retained in the final spectrum. Therefore, performing 1D first can avoid excessive noise amplification. By comparing these individually applied and combined preprocessing approaches, we comprehensively evaluated their performance across different scenarios. This process ultimately led to the identification of the most suitable preprocessing scheme for the requirements of this research. This work not only optimized the spectral data preprocessing pipeline but also laid a solid foundation for subsequent analysis, ensuring the reliability and validity of the research findings.

### 2.4 Development of prediction models

To comprehensively evaluate the predictive capability of near-infrared spectroscopy data for protein content in maize powder, this study constructed four distinct prediction models based on different principles. These included both traditional machine learning models and deep learning models. The specific models developed were Partial Least Squares Regression (PLSR), Support Vector Machine Regression (SVM), Residual Network (ResNet-18), and Transformer. By comparing the performance of these different models in predicting the protein content of maize powder, the study aims to identify the most suitable analytical method for the spectral data of maize powder.

#### 2.4.1 Partial least squares regression (PLSR)

Partial Least Squares Regression (PLSR) is a powerful and classical statistical method. It is also the most commonly used and widely applied multivariate linear regression technique for developing quantitative models in near-infrared spectroscopy analysis (24–26). This method is suitable for analyzing multivariate data, particularly when the independent variables are highly correlated (multicollinearity) or when the number of variables exceeds the number of samples. PLSR works by identifying the directions of maximum covariance between the spectral data matrix X and the protein content vector Y. It then establishes a linear regression model based on these latent variables. This approach effectively handles high-dimensional spectral data with multicollinearity. When extracting components, PLSR not only considers the structure of the independent variables but also their correlation with the dependent variable. This enhances the predictive performance of the model.

#### 2.4.2 Support vector machine (SVM)

The Support Vector Machine (SVM) is one of the most commonly used methods in spectral analysis (27, 28). Support Vector Regression (SVR) is a regression method based on SVM and can be divided into linear and non-linear types. To comprehensively compare the performance of linear and non-linear models, this study constructed both a Linear Kernel Support Vector Regression (Linear-SVR) model and a non-linear Radial Basis Function Kernel Support Vector Regression (RBF-SVR) model. SVM employs the kernel trick to map linearly inseparable low-dimensional data into a high-dimensional feature space, where linear regression is then performed. Specifically, the Linear-SVR model seeks the optimal linear separating hyperplane directly in the original feature space, making it suitable for modeling potential linear relationships. In contrast, the RBF-SVR model handles more complex, non-linear relationships through a non-linear mapping. Both models work by constructing an epsilon-insensitive tube around the data points to minimize prediction error. Their excellent generalization capability makes them particularly well-suited for processing high-dimensional spectral data.

#### 2.4.3 Deep learning algorithms

**Figure 3:**
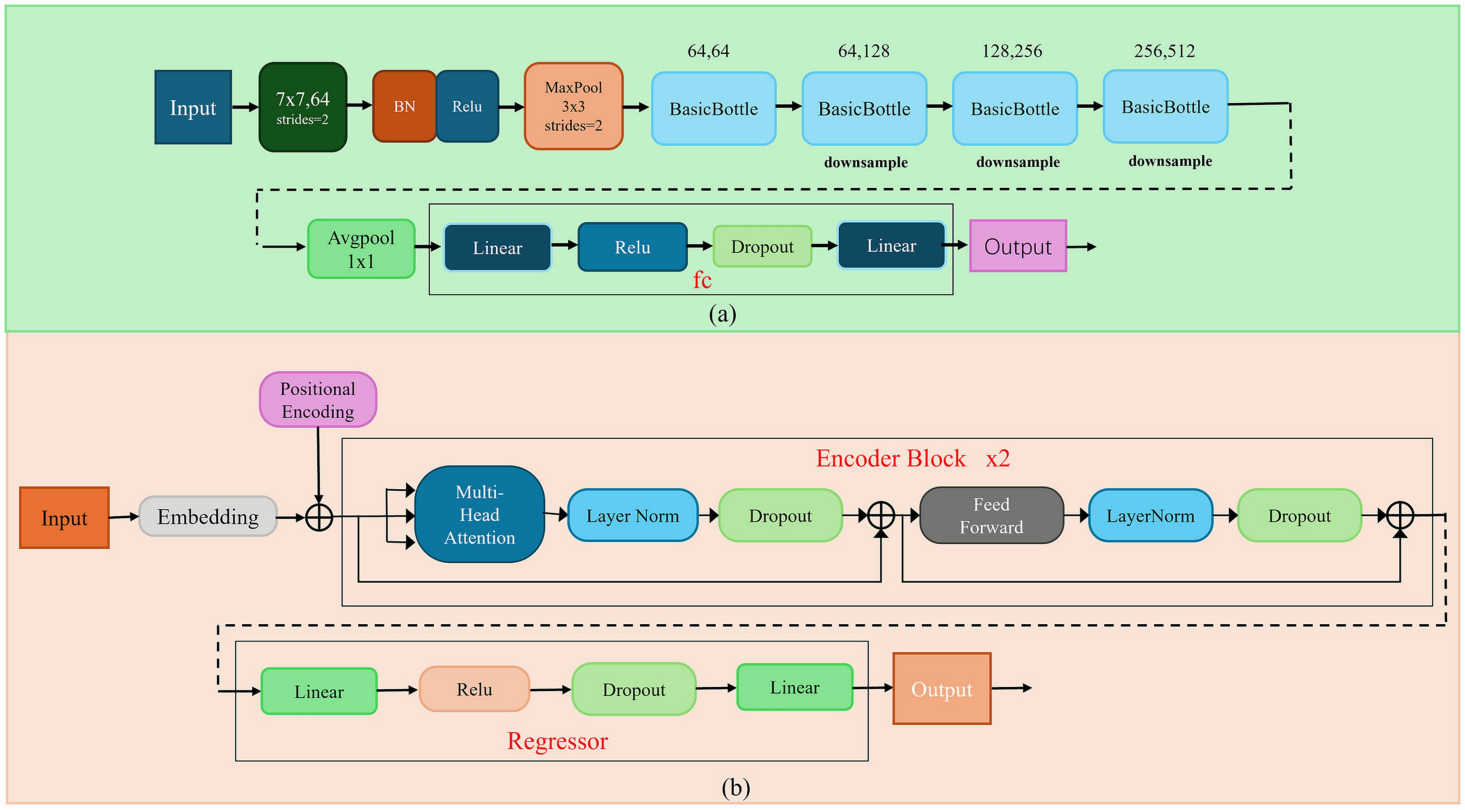
Architecture of the deep learning-based protein content prediction models. **(a)** ResNet-18; **(b)** transformer.

With the continuous development and optimization of deep learning algorithms, their application in spectral data analysis has become increasingly widespread. Considering the characteristics of maize powder spectral data, such as high dimensionality and the presence of baseline drift, this study selected two deep learning algorithms to construct detection models for protein content in maize powder: Residual Network (ResNet-18) and Transformer (29). The architectures of both models are shown in Figure 3.

The Residual Network (ResNet-18) model designed in this study can automatically extract deep-level spectral features. The model consists of convolutional layers, pooling layers, residual blocks, and fully connected layers. Initially, a 7×7 convolutional layer and a max-pooling layer perform rapid downsampling and preliminary feature extraction from the input spectrum. Subsequently, multiple stacked residual blocks, through their internal convolutional operations and skip connections, progressively extract deeper and more abstract spectral features. Finally, a global average pooling layer compresses the features into a fixed-length vector, which is then fed into a fully connected layer to output the predicted protein content. This end-to-end learning approach reduces excessive reliance on manual feature engineering (30).

To explore the application of state-of-the-art sequence modeling techniques in spectral analysis, this study also introduced the Transformer architecture (31). The Transformer model treats the preprocessed near-infrared spectrum as a sequence of wavelength points. Leveraging the global context modeling capability of its self-attention mechanism, it can capture long-range dependencies between different wavelength points across the entire spectral sequence. This allows for a more comprehensive analysis of the complex mapping between spectral features and protein content. The designed Transformer-based protein regression model comprises an embedding layer, a positional encoder, encoder layers, and a regressor. First, the embedding layer maps the absorbance value of each wavelength point into a high-dimensional vector, while the positional encoder injects sequential information. Then, multiple encoder layers, through their core multi-head self-attention mechanisms and feed-forward neural networks, globally capture complex dependencies among wavelength points. Finally, the regressor maps the aggregated sequence information into the predicted protein content value.

### 2.5 Feature selection

In the field of spectral data analysis, feature selection is a crucial step. Its core objective is to accurately identify the most informative features from a large number of spectral variables. This process plays a vital role in improving model interpretability and predictive accuracy. Among the various feature selection methods, Partial Least Squares Regression Coefficients (PLSRC), Competitive Adaptive Reweighted Sampling (CARS), Successive Projections Algorithm (SPA), and Uninformative Variable Elimination (UVE) are widely used.

Partial Least Squares Regression Coefficients (PLSRC) is a feature selection method based on regression analysis. It evaluates the contribution of each variable to the model by calculating its regression coefficient. A larger absolute value of the regression coefficient indicates a higher importance of that variable to the model. This method allows for the direct extraction of key variables from the regression model, providing strong support for model simplification and optimization. Competitive Adaptive Reweighted Sampling (CARS) is a feature selection method based on sampling techniques. It employs multiple rounds of random sampling to progressively select the variables that contribute the most to the model’s predictive ability. The CARS method performs exceptionally well when handling high-dimensional data, effectively reducing the number of variables while maintaining the model’s predictive accuracy.

The Successive Projections Algorithm (SPA) is a feature selection method based on variable projection. It identifies the most valuable variables for model prediction by projecting them into a lower-dimensional space. When dealing with complex spectral data, this method can effectively reduce data dimensionality and improve computational efficiency. Uninformative Variable Elimination (UVE) is a feature selection method based on variable importance assessment. It calculates an importance index for each variable and iteratively eliminates those that contribute less to the model.

These feature selection methods each have their own advantages and play important roles in different application scenarios. Through the appropriate selection and application of these methods, it is possible to effectively suppress interference from irrelevant noise and variables. This enhances the model’s predictive robustness and interpretability, significantly improving the efficiency and accuracy of spectral data analysis.

### 2.6 Model evaluation

This study selected three performance metrics for model evaluation: the correlation coefficient (R), the root mean square error (RMSE), and the residual predictive deviation (RPD). Larger RPD and R values, along with a smaller RMSE value, indicate higher regression accuracy of the model. The specific calculation formulas for these evaluation metrics are as follows (Equations 5–7):


$$\begin{array}{c} R=\frac{\sum_{i=1}^{n}(x_{i}-\bar{x})(y_{i}-\bar{y})}{\sqrt{\sum_{i=1}^{n}{\left(x_{i}-\bar{x}\right)}^{2}*\sum_{i=1}^{n}{\left(y_{i}-\bar{y}\right)}^{2}}} \end{array}$$ (5)

$$\begin{array}{c} \text{RMSE}=\sqrt{\frac{1}{m}\sum_{i=1}^{m}{\left(y_{i}-\bar{y}\right)}^{2}} \end{array}$$ (6)

$$\begin{array}{c} \mathrm{RPD}=\frac{\mathrm{SD}}{\mathrm{RMS}E_{P}} \end{array}$$ (7)

## 3 Results

### 3.1 Dataset partitioning for prediction models

This study collected spectral information and protein content data from a total of 90 maize powder samples. The protein content range was 8.43–11.25%, with an average protein content of 9.864%. Considering the characteristics of the maize powder spectral data, the SPXY method was employed to partition the complete dataset into a calibration set and a validation set using a 2:1 ratio. The SPXY method was chosen because it simultaneously maximizes the distances in both the spectral space (x) and the response variable space (protein content y) during the partitioning process. This approach ensures both diversity in spectral profiles and uniform coverage of the protein content range. The specific partitioning results are shown in Table 1.

**Table 1:** Reference component statistics for the calibration set and validation set of samples.

Sample set	Number of samples	Protein range (%)	Mean protein content (%)	Standard deviation
Calibration Set	60	8.430–11.250	9.781	0.744
Validation Set	30	8.865–11.150	10.031	0.639

### 3.2 Near-infrared spectral analysis

**Figure 4:**
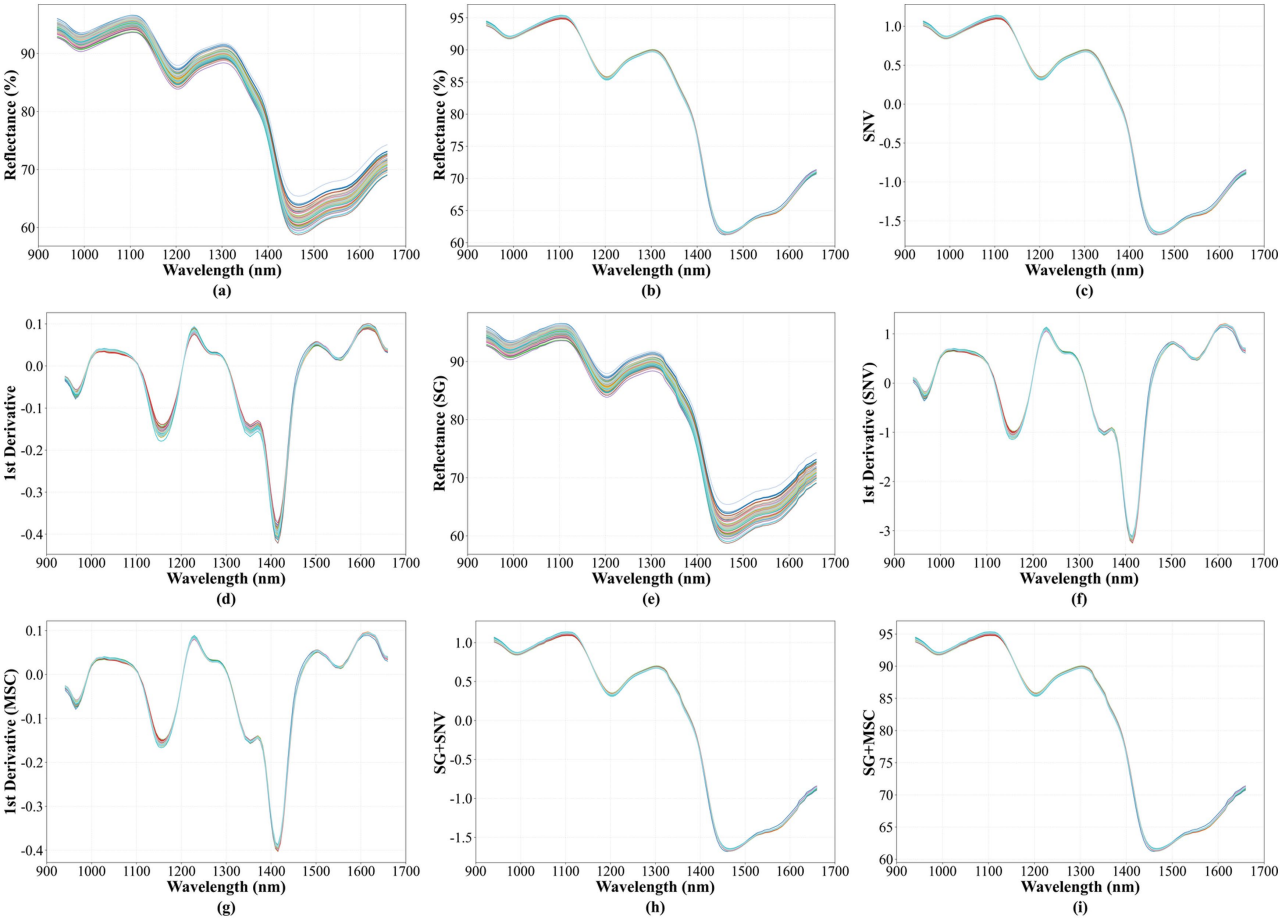
Preprocessed Spectra. **(a)** Untreated original spectra; **(b)** spectra processed using Multiplicative Scatter Correction (MSC); **(c)** spectra processed using Standard Normal Variate (SNV); **(d)** spectra processed using First Derivative (1D); **(e)** spectra processed using Savitzky–Golay (SG) smoothing; **(f)** spectra processed using a combination of 1D and SNV; **(g)** spectra processed using a combination of 1D and MSC; **(h)** spectra processed using a combination of SG and SNV; **(i)** spectra processed using a combination of SG and MSC.

Figure 4 displays the spectral curves of all collected maize powder samples within the wavelength range of 940 nm to 1,660 nm. Figure 4a shows the original spectral curves of the maize powder samples. The average spectrum of three measurement points for each sample constitutes its original spectral curve. The graph indicates that the overall trends of the spectral curves for the selected samples are largely consistent, with no anomalous abrupt changes observed. This consistency suggests that the selected maize powder samples are appropriate for the study.

Near-infrared spectroscopy (NIRS) is an analytical technique based on molecular vibrational energy level transitions. Its signals primarily originate from the absorption of infrared light by X-H bonds, including C-H, O-H, and N-H bonds (32). The intensity and position of absorption peaks exhibit specific differences depending on the type of hydrogen-containing functional group and its chemical environment. Analysis of the spectral data and curves of the maize powder revealed significant absorption peaks at approximately 1,000, 1,206, and 1,460 nm. The absorption peak near the 1,000 nm band is attributed to the second overtone of the N-H bond stretching vibration (33, 34). The peak near the 1,206 nm band is related to the second overtone of the C-H bond stretching vibration (35). The absorption peak around 1,460 nm originates from the first overtone of the O-H bond stretching vibration (36, 37). The chemical group information corresponding to these absorption peaks is consistent with characteristic structures such as -NH, -NH₂, and -COOH, which are found in protein molecules. This indicated that the NIRS data effectively reflect information about protein-related structures in the maize powder.

However, the spectral curves exhibit noticeable baseline drift and amplitude variation. Significant scattering effects between different samples can also adversely affect subsequent modeling. To address issues like baseline drift and amplitude differences, this study selected several preprocessing methods to optimize the original spectral curves. These methods included Multiplicative Scatter Correction (MSC), Standard Normal Variate (SNV), First Derivative (1D), and Savitzky–Golay (S–G) smoothing. The results are shown in Figure 4. From the figure, it is evident that both MSC and SNV significantly reduced baseline drift and amplitude variation. They eliminated multiplicative scattering while preserving the characteristic spectral shape. The 1D preprocessing eliminated baseline drift and accentuated absorption peaks and valleys. However, it also amplified some noise, resulting in rougher curves. The S–G smoothing preprocessing primarily removed some noise, so the curves did not show drastic changes compared to the original spectra.

To overcome the limitations of single preprocessing methods, such as noise amplification by the first derivative, this study attempted to combine multiple methods to leverage their respective advantages. Specifically, MSC or SNV were combined with 1D and S–G smoothing. These combined strategies aimed to simultaneously eliminate baseline drift and amplitude differences, suppress random noise, and enhance feature information like absorption peaks and valleys. As the effectiveness of different preprocessing steps is difficult to judge solely from the spectral curves, prediction models based on full-wavelength spectral data were developed to provide an objective evaluation. The predictive performance of these models was compared to objectively evaluate the effectiveness of the different preprocessing methods and the models themselves.

### 3.3 Analysis of prediction results

#### 3.3.1 PLSR prediction model

Following the spectral curve analysis, four protein content prediction models based on full-wavelength spectra were constructed using the calibration and validation sets detailed in Table 1. Initially, full-wavelength prediction models employing the PLSR algorithm were established using the eight preprocessed spectral datasets. The optimal number of latent components (n_components), which represents the number of score vectors the original high-dimensional spectra are compressed into, is critical. Setting this number too low can lead to underfitting, while setting it too high can cause overfitting. Therefore, the optimal number of latent components was determined through cross-validation, testing a range from 0 to 20. The final experimental results are presented in Table 2.

**Table 2:** PLSR model results based on different preprocessing methods.

Preprocessing methods	Optimal n_components	*R*_c_	RMSE_c_	*R*_p_	RMSE_p_	RPD
MSC	11	0.955	0.201	0.863	0.395	1.98
SNV	11	0.955	0.201	0.863	0.395	1.978
1D	6	0.931	0.246	0.876	0.385	2.031
SG	12	0.945	0.221	0.877	0.386	2.025
1D + SNV	5	0.917	0.27	0.873	0.382	2.145
1D + MSC	6	0.942	0.227	0.892	0.347	2.242
SG + SNV	13	0.945	0.221	0.863	0.398	1.964
SG + MSC	13	0.945	0.221	0.864	0.397	1.969

Table 2 compares the performance of the PLSR model under different preprocessing methods. Significant differences are observed in the goodness-of-fit metrics (*R*_c_, *R*_p_), root mean square errors (RMSE_c_, RMSE_p_), and predictive discrimination (RPD) across the various preprocessing techniques. The experimental data show that *R*_c_ values primarily range between 0.917 and 0.955, while *R*_p_ values mainly fall between 0.863 and 0.892. This indicates a generally good overall predictive performance. The combined 1D + MSC preprocessing method yielded the best results. It achieved values of 0.892 for *R*_p_, 0.347 for RMSE_p_, and 2.242 for RPD. This demonstrates that combining the first derivative with multiplicative scatter correction effectively removes scattering and baseline drift, thereby enhancing the correlation between the spectra and the target variable. Furthermore, an RPD value greater than 2 indicates that the model possesses good predictive capability. The 1D + SNV combination performed second best, with an RPD of 2.145. In contrast, the combined SG + MSC and SG + SNV methods resulted in decreased performance. This suggests that excessive smoothing during preprocessing may lead to the loss of critical spectral information.

#### 3.3.2 SVM prediction model

Linear kernel SVR (Linear-SVR) and radial basis function kernel SVR (RBF-SVR) models were established using the same eight pre-processed spectral datasets. The penalty parameter (C), the RBF kernel width (gamma), and the epsilon-insensitive band (epsilon) in SVR are crucial parameters for the SVR model. In the experiments, C was tested at values of 0.01, 0.1, 1, 10, and 100; gamma was tested at 0.0001, 0.001, 0.01, 0.1, 1, and 10; and epsilon was tested at 0.01, 0.05, 0.1, and 0.2. Cross-validation was performed for different combinations of these parameters. The final experimental results are shown in Tables 3, 4.

**Table 3:** Linear-SVR model results based on different preprocessing methods.

Preprocessing methods	*C*	Gamma	Epsilon	*R*_c_	RMSE_c_	*R*_p_	RMSE_p_	RPD
MSC	1	/	0.01	0.938	0.248	0.879	0.331	2.212
SNV	1		0.01	0.938	0.248	0.876	0.336	2.198
1D	0.01		0.05	0.917	0.286	0.848	0.385	1.869
SG	10		0.1	0.915	0.289	0.877	0.357	2.039
1D + SNV	0.01		0.01	0.92	0.284	0.844	0.389	1.811
1D + MSC	0.01		0.01	0.91	0.305	0.862	0.373	1.895
SG + SNV	1		0.05	0.917	0.285	0.878	0.339	2.19
SG + MSC	1		0.05	0.917	0.285	0.878	0.339	2.193

**Table 4:** RBF-SVR model results based on different preprocessing methods.

Preprocessing methods	*C*	Gamma	Epsilon	*R*_c_	RMSE_c_	*R*_p_	RMSE_p_	RPD
MSC	1,000	0.0001	0.05	0.906	0.3	0.854	0.371	1.934
SNV	1,000	0.0001	0.05	0.906	0.3	0.854	0.371	1.935
1D	100	0.0001	0.1	0.938	0.25	0.874	0.366	1.987
SG	1,000	0.001	0.2	0.867	0.354	0.691	0.509	1.398
1D + SNV	100	0.0001	0.01	0.943	0.245	0.85	0.377	1.863
1D + MSC	100	0.0001	0.01	0.949	0.207	0.822	0.411	1.73
SG + SNV	1,000	0.0001	0.05	0.9	0.31	0.852	0.375	1.912
SG + MSC	1,000	0.0001	0.05	0.9	0.31	0.852	0.375	1.912

Tables 3, 4 compare the performance of Linear-SVR and non-linear RBF-SVR models under different preprocessing methods. The results indicate that the Linear-SVR model combined with Multiplicative Scatter Correction (MSC) preprocessing achieved the best predictive performance (*R*_p_ = 0.879, RMSE_p_ = 0.331, RPD = 2.212). The Linear kernel model generally outperformed the RBF kernel model, suggesting a strong linear relationship exists between the spectral data and the target variable. The superior performance of Linear-SVM may stem from the inherent characteristics of the spectral data, where an approximately linear relationship exists between the spectral data (absorbance values) and protein content. In contrast, the RBF kernel model may have suffered from overfitting due to its excessive complexity.

#### 3.3.3 ResNet-18 and transformer prediction models

Prediction models for protein content were developed using ResNet-18 and Transformer architectures, respectively, each combined with the same eight different preprocessing methods. To ensure the reliability of the comparative experiments, both deep learning algorithms were compared under identical experimental conditions, including the same dataset, optimizer, loss function, learning rate scheduler, and number of training epochs. Based on multiple preliminary experiments, the final parameters were set as follows: learning_rate = 0.001, weight_decay = 0.0005, and batch_size = 128. Preliminary experiments also indicated that the models converged when the number of epochs exceeded 150. Consequently, the training iteration count was set to 150. Additionally, the Adaptive Moment Estimation (Adam) optimizer was selected for both models, and the Mean Squared Error Loss (MSE Loss) was used as the loss function. The final prediction results for the two models are presented in Tables 5, 6, respectively.

**Table 5:** ResNet-18 model results based on different preprocessing methods.

Preprocessing methods	*R*_c_	RMSE_c_	*R*_p_	RMSE_p_	RPD
MSC	0.965	0.195	0.647	0.514	1.242
SNV	0.962	0.206	0.53	0.582	1.097
1D	0.963	0.2	0.767	0.446	1.432
S–G	0.895	0.308	0.633	0.534	1.165
1D + SNV	0.967	0.187	0.669	0.496	1.288
1D + MSC	0.949	0.234	0.645	0.514	1.242
SG + SNV	0.969	0.185	0.587	0.579	1.103
SG + MSC	0.956	0.219	0.596	0.549	1.163

**Table 6:** Transformer model results based on different preprocessing methods.

Preprocessing methods	*R*_c_	RMSE_c_	*R*_p_	RMSE_p_	RPD
MSC	0.88	0.352	0.774	0.413	1.489
SNV	0.896	0.328	0.76	0.43	1.433
1D	0.952	0.243	0.827	0.351	1.754
S–G	0.856	0.379	0.732	0.477	1.287
1D + SNV	0.949	0.234	0.863	0.321	1.918
1D + MSC	0.944	0.246	0.848	0.337	1.828
SG + SNV	0.875	0.359	0.778	0.409	1.506
SG + MSC	0.862	0.376	0.752	0.426	1.447

Tables 5, 6 compare the performance of the ResNet-18 and Transformer models, respectively, under different preprocessing methods. From Table 5, it can be observed that for the ResNet-18 model, 1D preprocessing yielded the best performance (*R*_c_: 0.963, *R*_p_: 0.767, RPD: 1.432), once again demonstrating the importance of derivative information. This was followed by the two combined preprocessing methods: 1D + SNV and 1D + MSC. From Table 6, for the Transformer model, the combination of 1D + SNV proved most effective, achieving *R*_c_ and RMSE_c_ values of 0.949 and 0.234, respectively, and *R*_p_, RMSE_p_, and RPD values of 0.863, 0.321, and 1.918, respectively. The 1D + MSC combination was the next best performer (*R*_p_: 0.848, RMSE_p_: 0.337, RPD: 1.828). Notably, both the 1D + SNV and 1D + MSC combined preprocessing methods performed well in the two deep learning models. Furthermore, comparing the two models reveals that the Transformer model generally outperformed the ResNet-18 model across different preprocessing methods. This suggests that the Transformer architecture is more effective at utilizing spectral features to capture variations related to protein content.

This study systematically compared the performance of four different models combined with eight preprocessing methods for spectral data analysis. The results indicate that the combined preprocessing methods involving First Derivative (1D) with MSC or SNV generally performed best, achieving the top results in most models. This superiority stems mainly from the synergistic effect of these preprocessing steps: MSC/SNV effectively eliminates physical interference caused by particle size and scattering effects, standardizing the spectra to a common baseline. Subsequent 1D processing further removes residual baseline drift, enhances spectral resolution, and accentuates characteristic absorption peaks related to protein functional groups. This processing pipeline maximizes the extraction of spectral features directly relevant to protein content, thereby providing higher-quality input data for subsequent quantitative calibration models.

Additionally, the traditional methods, PLSR and Linear-SVM, generally outperformed the deep learning models, Transformer and ResNet-18. Although the Transformer’s performance was sufficient for protein content prediction, overfitting was observed with some preprocessing methods, and this issue was more pronounced in the ResNet-18 model. This is likely because NIRS data typically comprises several dozen to a few hundred samples, each with hundreds or even thousands of wavelength points, making it a classic high-dimensional, small-sample-size dataset. However, the substantial parameter complexity of deep learning models requires large-scale datasets for stable training. With small-sample-size dataset, these models are prone to memorizing dataset-specific noise and idiosyncratic features from the calibration set, rather than extracting generalizable spectral patterns. This fundamental mismatch between model complexity and data volume directly leads to the observed performance gap and overfitting. In contrast, traditional methods such as PLSR and Linear-SVM demonstrated superior performance. This advantage likely stems from the inherent characteristics of near-infrared spectroscopy (NIRS) data, which typically exhibit high collinearity among wavelength variables and a strong linear relationship between spectral response and the target property. These data characteristics align well with the underlying assumptions of traditional methods like PLSR and Linear-SVM, enabling them to construct predictive models more efficiently and robustly, thereby demonstrating enhanced generalization capability. Meanwhile, compared to deep learning algorithms, machine learning algorithms can build models specifically for small sample sets. Machine learning algorithms typically have faster training speeds and lower resource demands than deep learning (38). This study confirms that in the field of spectral analysis, traditional methods still hold an advantage compared to deep learning.

The experimental results show that the PLSR model, combined with the 1D + MSC preprocessing method, performs better and is more stable than other approaches. Therefore, this combination was selected for further feature selection experiments. Traditional chemometric methods still hold significant advantages in processing spectral data. Given the current dataset size, traditional machine learning methods demonstrate greater practicality and reliability. In the future, more sample data will be collected. Alternatively, lighter deep learning models will be developed. These steps will help further explore the potential of deep learning algorithms in spectral data analysis.

### 3.4 Analysis of protein content prediction models based on feature-selected spectra

Multicollinearity in near-infrared spectral data can interfere with the accuracy of prediction models. Therefore, effectively selecting key spectral features is a crucial step toward improving model performance. Moreover, reducing the number of input variables when building practical models significantly enhances computational efficiency. This study applied four different feature selection methods to the raw spectral data: partial least squares regression coefficients (PLSRC), competitive adaptive reweighted sampling (CARS), successive projections algorithm (SPA), and uninformative variable elimination (UVE). These methods were used to precisely identify spectral regions most correlated with protein content. The aim was to reduce data dimensionality while maintaining reliable predictive performance, thereby increasing computational speed and system responsiveness. Based on the preceding analysis, the 1D + MSC preprocessing method combined with the PLSR modeling approach was selected for feature selection.

After applying the 1D + MSC preprocessing, this study systematically compared the modeling performance of PLSR combined with four feature selection methods. Results were also compared against the full-spectrum baseline model (without feature selection processing), which had a runtime of 0.192 s, as shown in Table 7. All models using feature selection showed improved computational speed compared to the full-spectrum model. Among the four methods, PLSRC selected the largest number of characteristic wavelengths, followed by CARS and UVE. SPA selected the fewest wavelengths. Notably, the SPA method performed significantly better than the others, showing the most substantial improvement. Its *R*_c_, RMSE_c_, *R*_p_, and RMSE_P_ values were 0.954, 0.206, 0.927, and 0.301, respectively. The RPD increased to 2.502, and computation time was reduced to 0.166 s. Compared to the full-spectrum baseline model, this represents a speed improvement of approximately 13.5%. The SPA-based model also demonstrated the strongest generalization ability. CARS and UVE ranked second, with very similar performance. Both were clearly superior to the full-spectrum model without feature selection. PLSRC performed slightly worse than the other three methods. However, its prediction accuracy remained within an acceptable range.

**Table 7:** Performance of different feature selection methods.

Feature selection method	Number of characteristic wavelengths	Optimal n_components	*R*_c_	RMSE_c_	*R*_p_	RMSE_p_	RPD	Time (s)
X	/	6	0.942	0.227	0.892	0.347	2.242	0.192
PLSRC	48	5	0.949	0.223	0.885	0.327	2.156	0.188
CARS	46	11	0.94	0.231	0.893	0.336	2.245	0.186
SPA	19	9	0.954	0.206	0.927	0.301	2.502	0.166
UVE	45	7	0.942	0.226	0.894	0.324	2.247	0.181

It is noteworthy that after employing SPA for feature selection, the optimal number of latent variables in the PLSR model increased from 6 (for the full-spectrum model) to 9. This change can be attributed to the following reasons: the full-spectrum data contained a large number of highly collinear wavelength variables. The PLSR algorithm could effectively capture the co-varying information within these variables using only a few latent variables. Furthermore, the full-spectrum data also included more noisy variables, and the PLSR model might have begun fitting to noise after a small number of LVs, leading to premature termination. In contrast, the feature wavelengths selected by SPA exhibited a high signal-to-noise ratio and low inter-variable redundancy, with each wavelength potentially carrying unique information about different functional groups or vibrational modes of proteins. Consequently, the model required more latent variables to fully explore and integrate these refined yet discrete key pieces of information, thereby constructing a more robust model with superior predictive ability. This result also indirectly confirms the advantage of SPA in eliminating redundancy and enhancing data quality. Therefore, the change in the optimal number of latent variables is reasonable.

**Figure 5:**
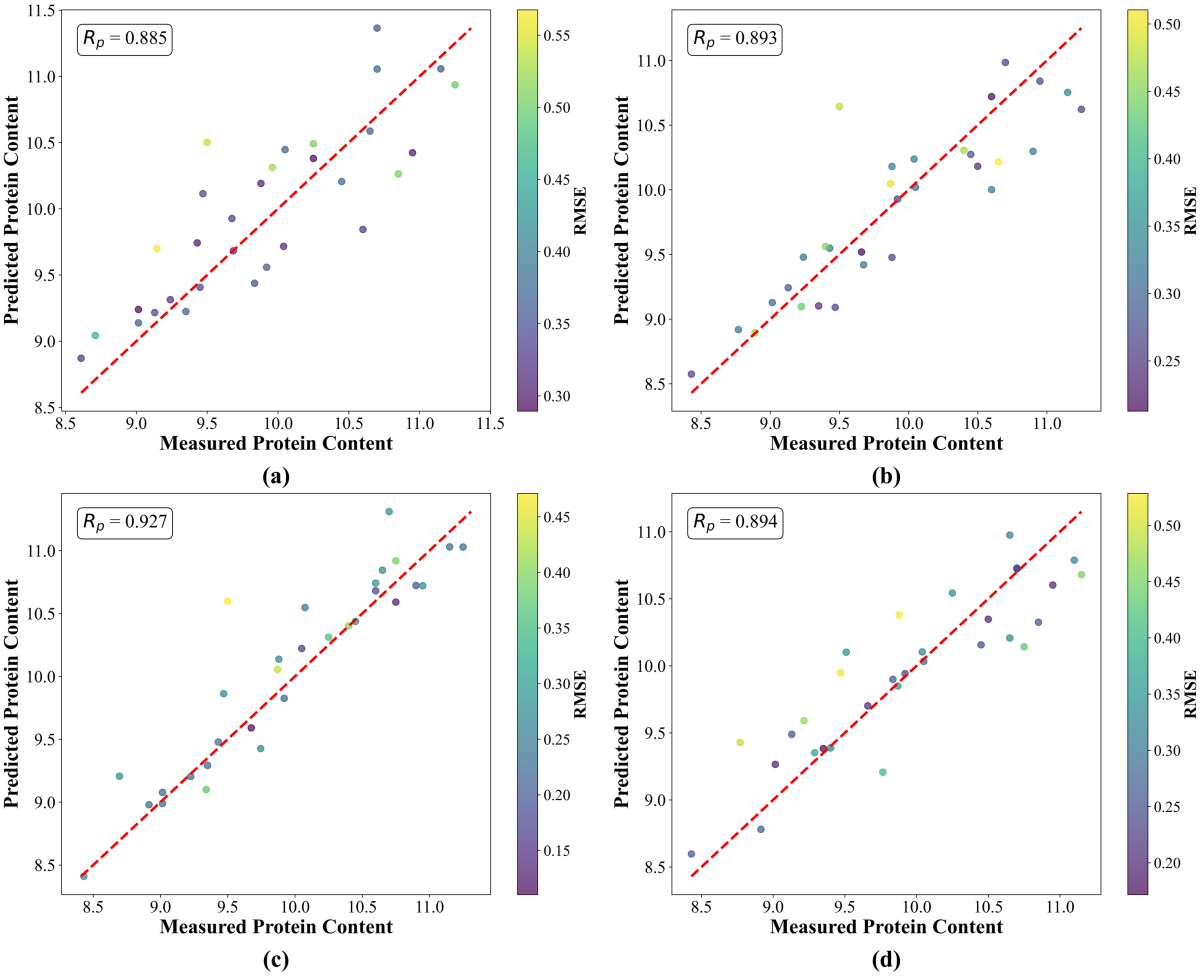
Prediction scatter plots using different feature selection methods: **(a)** PLSRC; **(b)** CARS; **(c)** SPA; **(d)** UVE.

Figure 5 shows the prediction scatter plots for protein content based on the four feature selection methods. Each point represents a sample’s actual value and its model-predicted value. The red line indicates the ideal prediction line. The closer the points lie to this line, the higher the prediction accuracy. Additionally, the color of each point indicates the magnitude of the prediction error. Compared with the other three methods, the prediction scatter plot for the SPA method has more points clustered around the ideal prediction line, and most points fall within the low-error range. This indicates that the model built using wavelengths selected by SPA achieves the best predictive performance and the lowest error.

**Figure 6:**
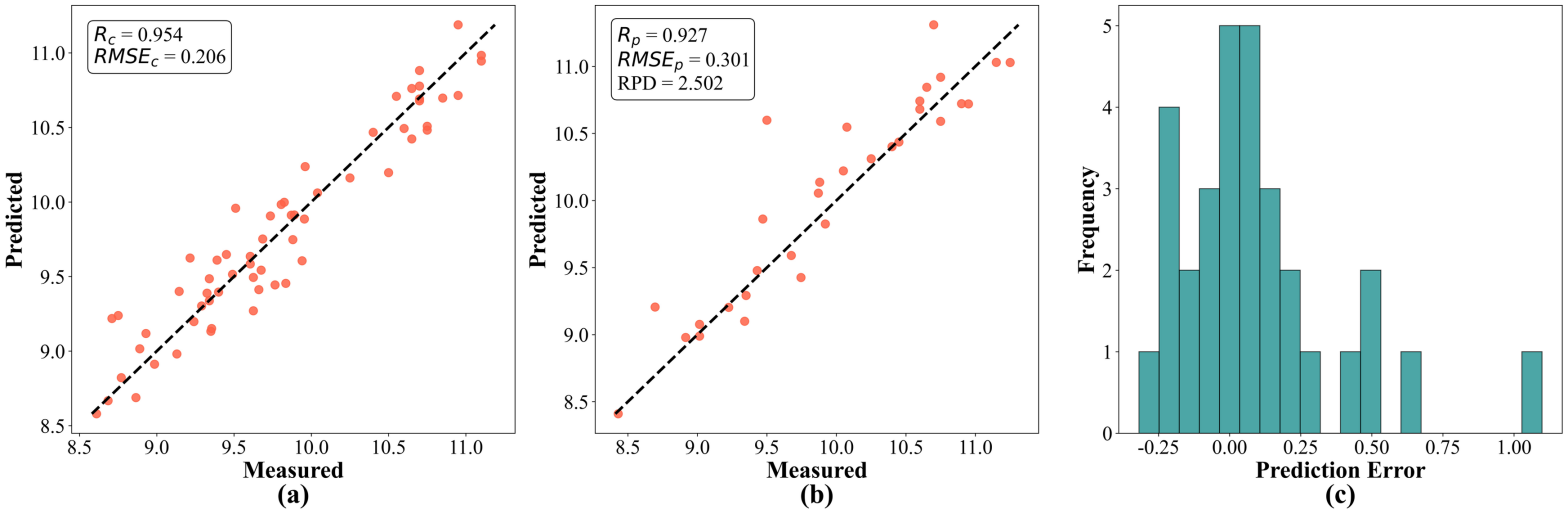
Prediction results of protein content based on the SPA feature selection method: **(a)** predictive scatter plot for the calibration set; **(b)** predictive scatter plot for the validation set; **(c)** histogram of prediction error distribution.

Figure 6 illustrates the prediction outcomes of protein content using the PLSR model combined with SPA feature selection and 1D + MSC preprocessing method. As shown, in the scatter plots (a) for the calibration set and (b) for the validation set, the predicted values closely follow the measured values around the 1:1 line without evident systematic bias across different concentration ranges. This observation indicates that the model provides a good linear response throughout the measurement range. The performance metrics for the validation set are close to those of the calibration set, suggesting that the model does not suffer from overfitting and possesses strong generalization capability. The high RPD value further confirms that this model is capable of accurate quantitative predictions and can distinguish between subtle concentration differences effectively. From the histogram of prediction error distribution (c), it is evident that the prediction errors for protein content fall within −0.25 to 1%, mostly concentrated within ±0.25%. This signifies minimal prediction errors. Additionally, the model requires low computational effort and operates rapidly, meeting practical application requirements. Therefore, by leveraging near-infrared technology and machine learning algorithms, this study has developed a high-performance protein content prediction model. It demonstrates excellent predictive accuracy and practical utility, providing a reliable technical solution for the rapid and non-destructive detection of protein content. This advancement is significant for improving the efficiency and reliability of quality control measures in the maize industry.

Corn powder was utilized as the detection matrix in this study. This approach aimed to eliminate the light scattering interference caused by surface irregularities and uneven protein distribution in whole-kernel corn samples, thereby enhancing the uniformity and reproducibility of the spectral signals. However, the grinding process itself may alter protein scattering behavior or induce conformational changes. Firstly, grinding modifies the scattering behavior of corn samples. Unlike the complex internal structural scattering in whole-kernel samples, the scattering behavior of ground powder is primarily governed by particle size and distribution. By controlling powder particle size through an 80-mesh sieve and applying MSC, SNV, and their combined preprocessing algorithms, this study effectively eliminated scattering effects induced by particle size differences (22, 39). Secondly, the mechanical shear forces and localized thermal effects associated with grinding may also lead to changes in protein conformation. These conformational alterations could affect the subtle shapes and intensities of near-infrared absorption peaks related to protein functional groups. Nevertheless, the quantitative model developed in this study demonstrated excellent predictive performance. This outcome indicates that under standardized grinding protocols, both the physically-induced scattering effects and potential conformational changes are systematic, consistent, and reproducible. Consequently, these effects are effectively calibrated by the model. The model also successfully established a reliable mapping relationship between stable spectral features of corn powder and protein content. This ensures the method’s validity and reliability for practical powder quality control applications in agricultural and processing industries.

Although the method proposed in this study demonstrates excellent performance in prediction accuracy and efficiency, it still has some limitations. Firstly, while the sample set used in this study covers major domestic production areas, its scale and diversity remain relatively limited for constructing a highly universal global model. Future research should include more samples of different varieties, vintages, and growing environments to further validate and enhance the model’s robustness and generalization ability. Secondly, although this study explores the role of deep learning methods in processing corn powder spectral data, the limitations in data volume may restrict the full performance potential of deep learning models. Future work will also focus on expanding the sample dataset and developing lightweight deep learning models to more deeply explore the potential of deep learning in spectral data analysis.

### 3.5 Analysis of feature selection results

To investigate the effectiveness of different feature selection methods in identifying relevant spectral information, this study further compared the selection outcomes of the four methods. The comparative results are shown in the figures.

**Figure 7:**
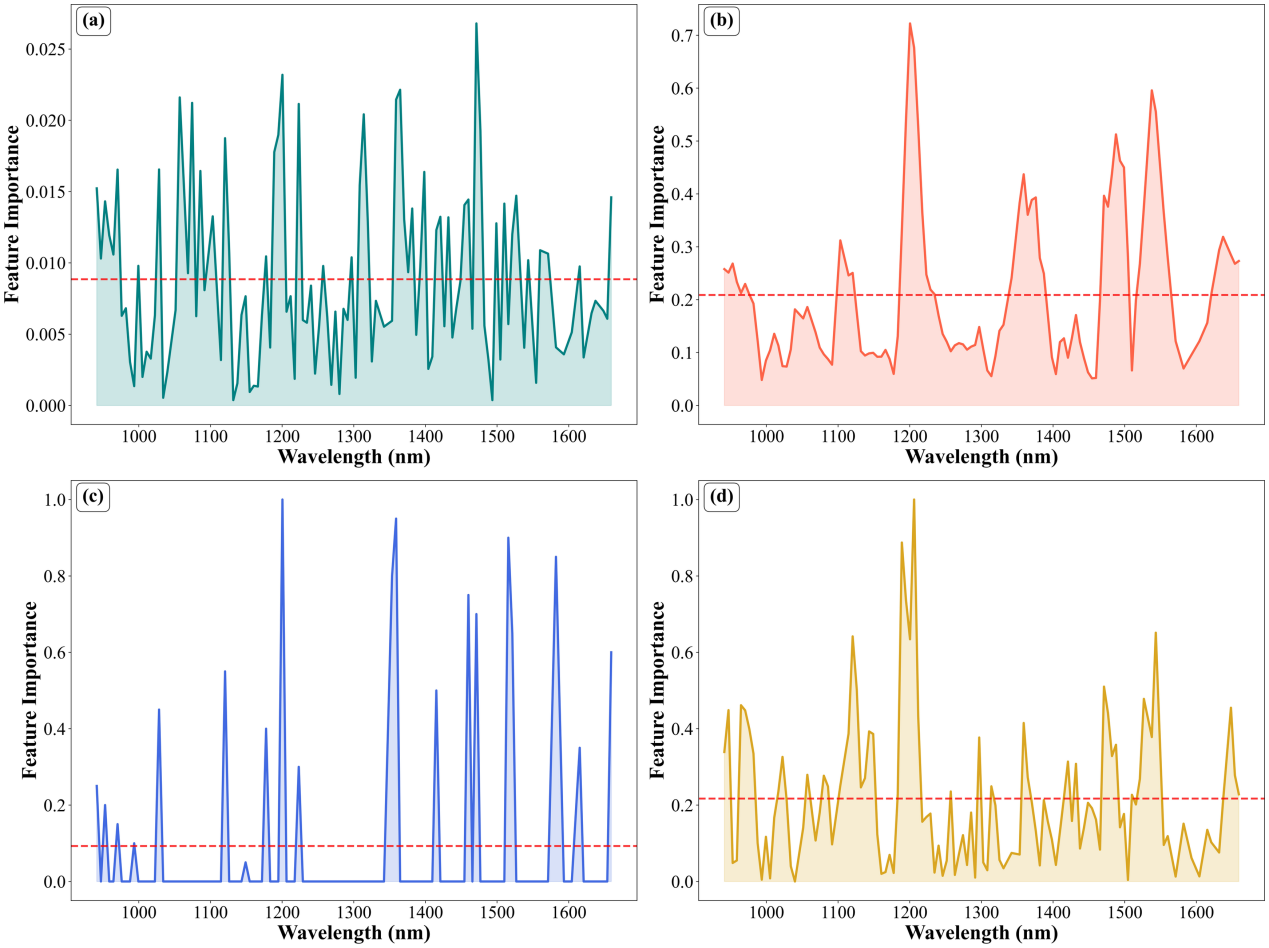
Feature importance analysis plots for the four selection methods: **(a)** PLSR; **(b)** CARS; **(c)** SPA; **(d)** UVE.

**Figure 8:**
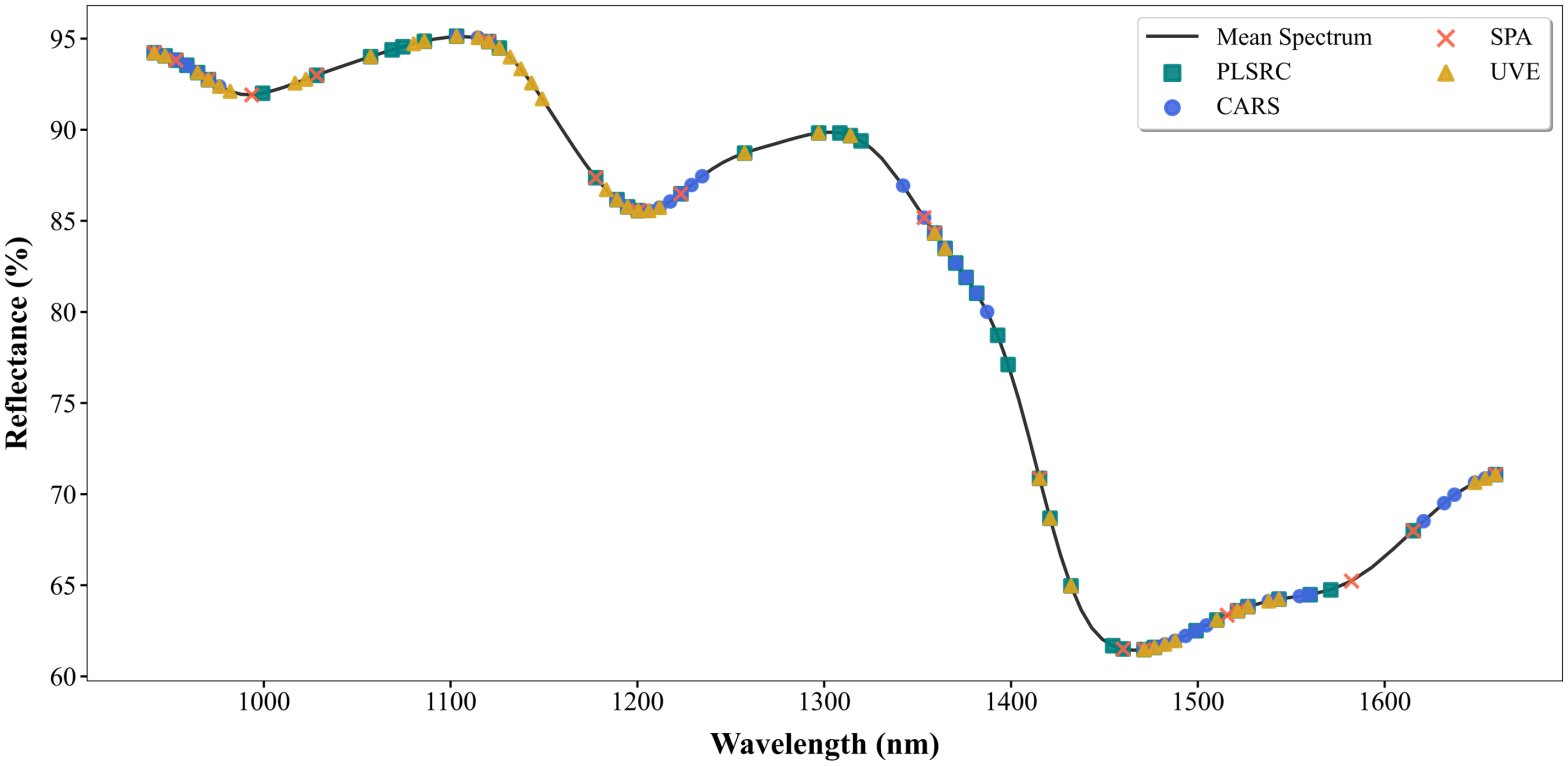
Distribution of characteristic wavelengths selected by the four feature selection methods.

Figure 7 displays the importance scores assigned by four different feature selection methods to the spectral bands, where a higher value indicates that the band is more important to the model. Figure 8 shows the distribution of the feature bands selected by the four feature selection methods—PLSRC, CARS, SPA, and UVE—plotted against the average spectral curve. From both figures, it can be observed that the four feature selection methods show general consistency in selecting most key regions. These regions correspond to the characteristic absorption of functional groups such as C–H and N–H in proteins. This indicates that all methods can identify spectral intervals closely related to protein structure and tend to select bands that are rich in information and high in discriminative power. However, differences remain in the specific strategies and selection outcomes of the different methods.

From Figure 7, it is evident that all four methods exhibit a clear tendency to select bands above the average importance level, demonstrating their effectiveness in identifying wavelengths with high informational content and significant contribution to model prediction. Specifically, the PLSRC method selected the largest number of features (48), showing relatively smooth and continuous peak distributions on the importance plot. However, it covered multiple continuous intervals such as 1,470–1,500 nm and 1,350–1,400 nm, resulting in high intra-region redundancy. The CARS and UVE methods yielded a moderate number of selected features (46 and 45, respectively). Their plots display multiple distinct peaks of varying heights, distributed relatively evenly, indicating a strong response to key regions while maintaining a certain breadth of spectral coverage. Notably, CARS selected several adjacent wavelengths around 1,200 nm (e.g., 1200.48 nm, 1206.18 nm, 1211.87 nm), exhibiting significant collinearity, which can reduce model stability and impair generalization ability. In contrast, the SPA method is notably different. It produced the smallest number of selected features (19), with importance scores highly concentrated on a few core bands, including key wavelengths such as 1200.48, 1359.10, 1515.71, and 1582.22 nm. Importantly, key wavelengths like 1515.71 nm (N–H absorption region) and 1582.22 nm (C–H combination band) were not selected by the other three methods. Furthermore, within the 1,460–1,600 nm interval, SPA retained only representative wavelengths like 1460.01, 1471.17, 1515.71, 1521.27, and 1582.22 nm. In comparison, PLSRC, CARS, and UVE selected numerous consecutive wavelengths in this region, introducing redundant information and causing information overlap. This outcome stems from SPA’s fundamental principle: it minimizes collinearity between variables by iteratively selecting the wavelength that maximizes the projection error, thereby constructing a minimally redundant yet highly representative feature subset. The minimal redundancy characteristic significantly enhances model performance through the following mechanisms. Firstly, it effectively suppresses overfitting by eliminating multicollinearity among variables, enabling the model to focus on discriminative spectral features rather than noise signals, thereby enhancing the model’s generalization capability. Secondly, this characteristic substantially improves computational efficiency. Since each wavelength in the feature subset carries complementary information without redundant contributions, the dimensionality of matrix operations during modeling is significantly reduced, leading to a notable increase in computational speed. This dual advantage enables the method to achieve synergistic optimization in both prediction accuracy and computational efficiency. This strategy enables the SPA to effectively mitigate information overlap and collinearity issues, allowing it to focus on wavelengths with truly high discriminative power (40, 41). Consequently, it achieves optimal performance in both predictive accuracy and computational speed.

Further analysis based on Figure 8 reveals that the wavelengths selected by PLSRC, CARS, and UVE cover broad spectral regions associated with protein absorption features. While comprehensive, their selection inevitably includes more intra-region redundancy and random redundancy. In contrast, the wavelengths selected by SPA are more refined and concentrated, clearly avoiding information-overlapping areas and corresponding precisely to a few core absorption peaks. These bands not only align accurately with characteristic protein functional groups but, more importantly, are mutually independent and contain complementary information. This selection mechanism contributes to its superior final modeling performance—achieving the highest *R*_p_, the lowest RMSE_p_, the best RPD value, and the fastest computational speed among the methods compared. Consequently, the SPA method not only controls data redundancy but also identifies feature wavelengths that are more closely aligned with the protein structure. This leads to higher predictive accuracy, faster computational speed, and stronger generalization capability.

## 4 Conclusion

This research successfully achieved accurate prediction of protein content in maize powder using near-infrared spectroscopy (NIRS) combined with machine learning algorithms. This study adopts powdered samples, instead of whole kernels, to effectively mitigate scattering and uneven distribution issues, leading to more stable spectra and superior prediction performance. Prediction models for protein content were developed based on both machine learning algorithms (PLSR, SVM) and deep learning algorithms (ResNet-18, Transformer), and their performances were compared using eight different preprocessing methods. The study found that the combined preprocessing methods involving the First Derivative (1D) with either SNV or MSC yielded the best results for processing the NIRS information. Traditional machine learning algorithms demonstrated an advantage over deep learning models for this spectral data analysis. This advantage stems from the high dimensionality and collinearity of the spectral variables, along with the approximately linear relationship between absorbance and protein concentration. These characteristics perfectly align with the underlying assumptions of traditional models. Furthermore, these conventional methods can build models tailored for small sample sizes, and they typically offer faster training speeds and lower computational resource demands. Among them, the PLSR model showed the best predictive performance, particularly when combined with the 1D + MSC preprocessing method. Furthermore, four different feature selection methods were employed to identify spectral wavelengths relevant to the protein content in maize powder. The feature selection process identified that the most predictive wavelengths were primarily located around 1,000 nm band (second overtone of N-H stretching), the 1,200 nm band (second overtone of C-H stretching), and the 1,500–1,600 nm region (encompassing N-H combination tones and C-H stretching overtones). A comparative analysis of the results indicated that the optimal protein content prediction model was achieved using the PLSR algorithm on 1D + MSC preprocessed data, combined with the Successive Projections Algorithm (SPA) for feature selection. The SPA method enhances model precision and efficiency by selecting key spectral features (e.g., 1,200 nm C-H, 1515 nm N-H bands) to build minimally redundant, low-collinearity wavelength sets. This optimal model achieved a correlation coefficient of prediction (*R*_p_) of 0.927, a root mean square error of prediction (RMSE_P_) of 0.301, and a residual predictive deviation (RPD) of 2.502. The prediction errors were concentrated within ±0.25%, and its computational speed surpassed that of the other three models. In practical production, this technology enables on-site rapid screening and quality grading of protein content in maize. It helps improve the quality control efficiency throughout the maize industry chain, reduces testing costs, and provides data support for precision agriculture and intelligent processing. Future research will include maize samples from different varieties and growing regions. Additionally, we are committed to establishing a more comprehensive standardized spectral library and developing spectral analysis network models characterized by reduced parameters and streamlined architectures. This initiative aims to further explore the potential of deep learning algorithms in spectral data analysis, ultimately enabling the creation of protein prediction models that integrate high precision, enhanced robustness, and practical utility.

## Data Availability

The original contributions presented in the study are included in the article/supplementary material, further inquiries can be directed to the corresponding author.
